# Umfrage zur Versorgungssituation von Patient:innen mit systemischem Lupus erythematodes und systemischer Sklerose mit Lungenbeteiligung in Rheinland-Pfalz und dem Saarland

**DOI:** 10.1007/s00393-026-01830-4

**Published:** 2026-06-09

**Authors:** Lennart Haßdenteufel, Kai Jahns, Ciaran Alberti, Andreas Schwarting, Matthias Dreher

**Affiliations:** 1 I. Medizinische Klinik und Poliklinik, Schwerpunkt Rheumatologie und klinische Immunologie, Universitätsmedizin der Johannes Gutenberg-Universität Mainz, Mainz, Deutschland; 2 RZ Rheumazentrum Rheinland-Pfalz GmbH, Bad Kreuznach, Deutschland; 3 Gastroenterologie, GPR Klinikum Rüsselsheim, Rüsselsheim am Main, Deutschland; 4 Universitäres Centrum für Autoimmunität, Universitätsmedizin der Johannes Gutenberg-Universität Mainz, Mainz, Deutschland

**Keywords:** Kollagenosen, Lungenbeteiligung, Epidemiologie, Therapie, Patient:innenversorgung, Collagenosis, Pulmonary involvement, Epidemiology, Therapy, Patient care

## Abstract

**Hintergrund:**

Kollagenosen wie systemischer Lupus erythematodes (SLE) und systemische Sklerose (SSc) erfordern aufgrund multisystemischer Manifestationen eine interdisziplinäre Versorgung. Insbesondere die pulmonale Beteiligung (CTD-ILD) ist prognostisch relevant. Daten zur realen Versorgungssituation sind jedoch limitiert, wodurch strukturelle Defizite schwer identifizierbar bleiben.

**Methodik:**

Mit einem selbstentwickelten 14-moduligen Fragebogen wurden zwischen 09/2024 und 03/2025 insgesamt 2173 Praxen verschiedener Fachrichtungen in Rheinland-Pfalz und dem Saarland kontaktiert. Erfasst wurden Versorgungsstrukturen, Patientenzahlen, pulmonale Manifestationen sowie pharmakologische Therapien. Die Auswertung erfolgte deskriptiv auf Behandler-Ebene.

**Ergebnisse:**

Insgesamt 67 Einrichtungen berichteten über 1569 Patienten (SLE: *n* = 859; SSc: *n* = 421), von denen 286 (≈18 %) eine Lungenbeteiligung aufwiesen. Die Versorgung erfolgt überwiegend in spezialisierten Zentren, jedoch mit relevanter Beteiligung des niedergelassenen Bereichs. Es zeigten sich deutliche Unterschiede in der Therapie: Während im ambulanten Sektor häufiger Glukokortikoide in höheren Dosierungen eingesetzt werden (bis ≈28 % > 10 mg/d), gelingt in Zentren durch vermehrten Einsatz von Biologika und krankheitsmodifizierenden Therapien, insbesondere Mycophenolat-Mofetil (MMF), eine signifikante Reduktion der Glukokortikoiddosis (≈1,5 % > 10 mg/d). MMF wird dabei vor allem in der rheumatologischen Versorgung deutlich häufiger eingesetzt als in anderen Fachrichtungen. Insgesamt bestehen Unterschiede im Einsatz leitliniengerechter Basistherapien sowie in der Symptomwahrnehmung.

**Diskussion:**

Die Ergebnisse zeigen relevante Versorgungsdefizite mit Übergewicht der Glukokortikoidtherapie und unzureichender Nutzung evidenzbasierter DMARDs wie MMF im niedergelassenen Bereich. Ursachen sind begrenzte rheumatologische Expertise, eingeschränkter Zugang zu spezialisierten Therapien und mangelnde interdisziplinäre Vernetzung. Eine Verbesserung erfordert den Ausbau rheumatologischer Strukturen, frühzeitige fachärztliche Anbindung sowie eine stärkere Implementierung leitliniengerechter, steroidsparender Therapiestrategien.

## Abstract (en)

**Background:**

Connective tissue diseases such as systemic lupus erythematosus (SLE) and systemic sclerosis (SSc) require interdisciplinary management due to their multisystem involvement. Pulmonary manifestations (CTD-ILD) are of particular prognostic relevance. However, real-world data on current healthcare provision remain limited.

**Methods:**

A cross-sectional survey (14 modules) was conducted between September 2024 and March 2025, targeting 2173 practices across various specialties in Rhineland–Palatinate and Saarland. Data on healthcare structures, patient numbers, pulmonary involvement, and pharmacological treatment were collected. Analysis was performed descriptively at the provider level.

**Results:**

A total of 67 institutions reported on 1569 patients (SLE: *n* = 859; SSc: *n* = 421), of whom 286 (≈18%) had pulmonary involvement. Care was predominantly delivered in specialized centers, but with substantial contribution from office-based physicians. Marked differences in treatment strategies were observed: office-based settings more frequently relied on higher-dose glucocorticoids (up to ≈28% > 10 mg/day), whereas specialized centers achieved significant glucocorticoid reduction (≈1.5% > 10 mg/day) through increased use of biologics and disease-modifying therapies, particularly mycophenolate mofetil (MMF). MMF was notably more frequently used in rheumatology-based care compared to other specialties.

**Conclusion:**

The findings reveal relevant deficiencies in care, particularly an overreliance on glucocorticoids and underutilization of evidence-based disease-modifying antirheumatic drugs (DMARDs) such as MMF in nonspecialized settings. Contributing factors likely include limited rheumatology expertise and insufficient interdisciplinary coordination. Improving care requires expansion of rheumatologic capacity, earlier specialist referral, and broader implementation of guideline-based, steroid-sparing treatment strategies.

Kollagenosen wie der systemische Lupus erythematodes (SLE) und die systemische Sklerose (SSc) können die unterschiedlichsten Organsysteme, wie beispielsweise die Lunge, befallen. Dementsprechend werden durch Patienten oft unterschiedliche Fachärzte aufgesucht, teils ohne eine feste rheumatologische Anbindung. Dieser Umstand erschwert ein systematisches Erfassen der aktuellen Versorgungssituation dieser Patienten im Versorgungsalltag. Um Ansatzpunkte für mögliche Therapieoptimierungen zu finden, ist es daher nötig, fachübergreifend einen Überblick über die Versorgungssituation zu gewinnen.

## Hintergrund und Fragestellung

Kollagenosen sind systemische Autoimmunerkrankungen, welche etliche Organe betreffen können. Zwei der häufigsten spezifischen Kollagenosen sind der systemische Lupus erythematodes und die systemische Sklerose [1].

Für Deutschland wird beim SLE und der SSc von einer Prävalenz zwischen 0,037 und 0,14 % bzw. 0,017–0,025 % und somit von bis zu 56.000 Betroffenen Erwachsenen ausgegangen [1, 2]. Von der SSc sind Frauen ca. 5‑mal häufiger betroffen als Männer. Bei den meisten Patienten manifestieren sich die ersten Symptome der SSc im Alter zwischen 40 und 60 Jahren, während etwa ein Viertel der Betroffenen bereits vor dem 40. Lebensjahr erkrankt. Der SLE zeigt ebenfalls eine deutlich höhere Inzidenz bei Frauen, die bis zu 9‑mal häufiger ist; dieser geschlechterspezifische Inzidenzunterschied ist besonders ausgeprägt im jüngeren Patientenalter zu beobachten [3–5].

Die interstitielle Lungenerkrankung (ILD) bezeichnet eine Gruppe diffuser parenchymaler Lungenerkrankungen, die durch Entzündung und/oder Fibrose des Lungeninterstitiums, dem Raum zwischen den einzelnen Alveolen, gekennzeichnet sind. Tritt eine ILD im Rahmen einer Kollagenose bzw. einer Bindegewebserkrankung („connective tissue disease“, CTD; diese beinhalten als Überbegriff auch Erkrankungen nichtentzündlicher Genese) auf, spricht man von einer sog. CTD-assoziierten ILD (CTD-ILD; [6]). Die Prävalenz hierfür wird in Deutschland mit 47 % bei der SSc und 6 % beim SLE beschrieben [6, 7].

Als Systemerkrankung ist der SLE in seiner Symptomatik äußert variabel [8]. Patienten mit SLE berichten am häufigsten über eine ausgeprägte Fatigue (> 90 %), gefolgt von Arthritiden (≈90 %), kutanen Manifestationen (≈80 %) und Fieber (≈78 %). In etwa 50 % der Fälle entwickelt sich zudem eine Lupusnephritis, die aufgrund der häufigen Progression zu einem sekundären chronischen Nierenversagen als verlaufs- und prognosebestimmender Faktor gilt [2, 9–13].

Während früher bei der SSc die Nierenbeteiligung (renale Krise) die Prognose bestimmte, ist es mittlerweile die Lungenfibrose bzw. ILD mit ca. 35 %, gefolgt von der pulmonalen Hypertonie und kardialen Ursachen mit jeweils ca. 26 % [14–16]. Weitere häufig auftretende Symptome der SSc sind neben der typischen kutanen Sklerose, die insbesondere an Fingern und Zehen auftritt (Sklerodaktylie), auch die Fatigue, das Raynaud-Phänomen, Arthralgien oder Myopathien sowie gastrointestinale Beschwerden [16].

Beim SLE gilt Hydroxychloroquin (HCQ), ein Antimalariamittel, als etablierte Basistherapie und wird bei allen SLE-Patienten empfohlen. Glukokortikoide (GC) bleiben gerade in der Akutphase zentral [17–20]. Um den langfristigen GC-Bedarf jedoch zu reduzieren, kommen Immunsuppressiva (IS) wie Azathioprin (AZA), Mycophenolat-Mofetil (MMF) oder Methotrexat (MTX) zum Einsatz. Zudem stehen als Zusatztherapien drei Biologika zur Verfügung: Belimumab (BEL; seit 2011), Anifrolumab (seit 2022) und Obinutuzumab (seit Ende 2025 bei der Lupusnephritis) [2, 19, 21].

Die Therapie der systemischen Sklerose (SSc) richtet sich primär nach den führenden klinischen Manifestationen. Zur Behandlung der vaskulären Komplikationen, insbesondere des Raynaud-Syndroms, akraler Ulzerationen sowie der pulmonalen Hypertonie, kommen vorrangig Vasodilatatoren zum Einsatz. Hierzu zählen Prostacyclin-Derivate, Kalziumkanalblocker, Endothelin-Rezeptorantagonisten und Phosphodiesterase-5-Inhibitoren. Für kutane und muskuloskeletale Manifestationen werden meist niedrig dosierte Glukokortikoide verabreicht, deren Nutzen jedoch kontrovers diskutiert wird. Häufiger werden krankheitsmodifizierende Therapien wie MTX, Rituximab (RTX) oder MMF eingesetzt. Bei myokardialer Fibrose finden Cyclophosphamid (CYC) und MMF therapeutische Anwendung [22–25]. Nintedanib (NIN) ist beim SLE nur bei einer progredienten ILD zugelassen.

Bei der SSC-ILD ist MMF die bevorzugte Behandlung (in Deutschland off-label) aufgrund seiner besseren Verträglichkeit und einer vergleichbaren Wirksamkeit im Vergleich zu CYC [26]. AZA wird hauptsächlich als Erhaltungstherapie nach einer intensiveren Behandlung eingesetzt [27]. Bei einer beginnenden SSc eignet sich zudem RTX als eine weitere Therapieoption, da ähnlich gute Effekte auf die Hautveränderungen und die Erhaltung der Lungenfunktion im Vergleich zu einer Therapie mit CYC gezeigt werden konnten [28]. Im Gegensatz zu Japan (zugelassen für die Indikation), ist RTX in den USA und Europa lediglich off-label einsetzbar. Ebenfalls steht im Off-label-Bereich Tocilizumab (TOZ) als wirksame Option zu Verfügung [29–31]. In Deutschland ist aktuell lediglich NIN bei SSc-ILD und der Lungenfibrose zugelassen [32, 33].

In besonders schweren und therapierefraktären Fällen zeigt eine nichtmyeloablativ durchgeführte autologe Stammzelltransplantation (AHSCT) eine langfristige Überlegenheit gegenüber Cyclophosphamid und stellt eine alternative Therapie dar [34–37]. Ebenso zeigen CAR-T-Zelltherapien in Fallserien eine gute klinische Wirksamkeit [38].

Trotz der erheblichen individuellen und gesellschaftlichen Belastung durch Kollagenosen wie die SSc und dem SLE fehlen belastbare Erkenntnisse zur Versorgungsrealität – insbesondere in Deutschland [39].

## Studiendesign und Untersuchungsmethoden

Die Datenerhebung erfolgte von September 2024 bis März 2025. Es wurden Fragebögen an Rheumatologen, Pneumologen, Nephrologen, Internisten ohne weitere Spezialisierung sowie Hausärzte in Rheinland-Pfalz und dem Saarland versendet.

Rheinland-Pfalz ist vorwiegend ländlich geprägt mit einigen städtischen Zentren (> 50.000 Einwohner). Größere städtische Zentren sind vorwiegend in Grenznähe zu anderen Bundesländern oder Nationen gelegen. Im Saarland hingegen liegt insgesamt eine hohe Bevölkerungsdichte vor, welche sich jedoch zum größten Teil in einem Ballungsraum konzentriert.

Die Umfrage erfolgte über drei verschiedene Kommunikationswege:

In der ersten Erhebungsphase (September bis Dezember 2024; Q4/2024) wurde der Fragebogen per E‑Mail an 1692 Ärztinnen und Ärzte versendet. In diesem Zeitraum gingen 114 vollständig ausgefüllte Fragebögen zurück, was einer Rücklaufquote von 6,7 % entspricht.

Im März 2025 erfolgte der Versand per Fax an 2168 Praxen gefolgt von einem postalischen Versand an 2173 Praxen, mit einem Rücklauf von weiteren 98 Fragebögen (9,6 % Rücklaufquote insgesamt). Die Empfänger wurden von Juni bis September 2024 über eine Internetrecherche getrennt nach Facharztrichtungen und Praxen bzw. Kliniken ermittelt.

Die Adressdaten der einzelnen Praxen (niedergelassene Fachärzte) und Zentren (Kliniken mit Ambulanzanbindung) wurden online über den KV-Praxisfinder und andere namhafte Portale wie Jameda oder medfuehrer.de recherchiert.

Für die vorliegende Studie wurde ein eigens entwickelter Fragebogen bestehend aus 14 Modulen, die einige Fragen zur Spezifikation der Praxis, der Anzahl von Kollagenosepatienten der Häufigkeit und Form der Lungenbeteiligung, der medikamentösen Versorgung dieser Patienten sowie einer Einschätzung der aktuellen Versorgungsituation von Kollagenosepatienten in Rheinland-Pfalz und dem Saarland beinhalten. Zusätzlich konnten in einem Freitextfeld Verbesserungsvorschläge genannt werden.

Zur Charakterisierung der teilnehmenden Versorger wurden strukturelle Merkmale erhoben, darunter das Versorgungssetting (niedergelassene Praxis vs. klinische Anbindung) sowie die jeweilige Fachrichtung (u. a. Rheumatologie, Allgemeinmedizin, Innere Medizin, Nephrologie, Pneumologie). Diese Angaben wurden mittels Multiple-Choice-Items erfasst.

Die Erhebung patientenbezogener Kennzahlen erfolgte in aggregierter Form auf Behandler-Ebene. Hierzu zählten die Anzahl der behandelten Patienten mit SLE und SSc, deren Geschlechterverteilung, das durchschnittliche Alter sowie die Häufigkeit einer Lungenbeteiligung. Diese Variablen wurden als metrische Größen in Form von Dezimalzahlen angegeben.

Symptome, Formen der Lungenbeteiligung sowie eingesetzte Therapien wurden als prozentuale Anteile bezogen auf die jeweilige Gesamtpopulation der Patienten mit SLE bzw. SSc und bestehender Lungenbeteiligung erhoben. Neben vorgegebenen Antwortkategorien bestand die Möglichkeit, zusätzliche Angaben über Freitextfelder zu ergänzen, um eine umfassendere Erfassung klinischer Variabilität zu gewährleisten.

Der Einsatz von Glukokortikoiden wurde ebenfalls prozentual erfasst, wobei vier vordefinierte Dosisintervalle (von kortisonfrei bis > 10 mg Prednisolonäquivalent pro Tag) zur Anwendung kamen.

Zur Beurteilung des Therapieerfolgs enthielt der Fragebogen ein Item zum Remissionsstatus der Patienten. Remission wurde hierbei einheitlich als das Fehlen von Krankheitsaktivität definiert. Ergänzend wurde der Anteil der Patienten erhoben, die als zufriedenstellend therapiert eingeschätzt wurden. Die Bewertung dieses Parameters erfolgte subjektiv durch die jeweiligen Behandler, um zusätzlich deren individuelle Einschätzung des Therapieerfolgs abzubilden.

Die Auswertung der Daten erfolgte deskriptiv, um einen Überblick über die Patienten mit Kollagenosen (SSc und SLE), insbesondere mit Lungenbeteiligung zu erhalten.

Für das Ausfüllen des Fragebogens konnte eine Aufwandsentschädigung bei Rücksendung des Fragebogens in Höhe von 30 € bei der Universitätsmedizinmedizin Mainz geltend gemacht werden; 28 (13,2 %) Behandler machten davon Gebrauch.

## Ergebnisse

Aus allen 3 Erhebungsphasen mit insgesamt 5 Erhebungsdurchgängen wurden 210 (9,6 % Rücklaufquote) Fragebögen empfangen und ausgewertet. Davon berichteten 67 Praxen, derzeit Patienten mit SLE oder SSc zu betreuen. Diese Praxen versorgen 1569 Kollagenosepatienten, von denen 284 eine pulmonale Beteiligung aufweisen.

**Abb. 1:**
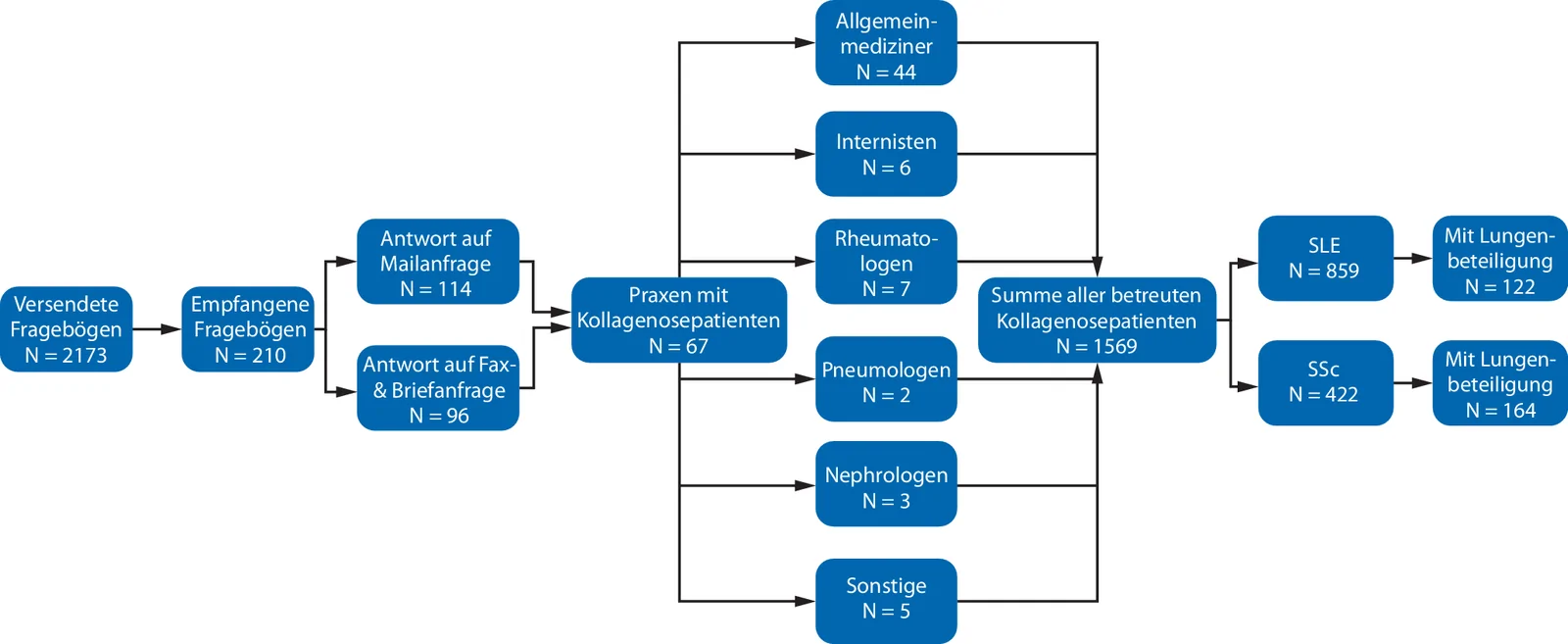
Verlaufsbaum der Versand- und Rücklaufquote der teilnehmenden Praxen und Zentren

Die Patienten verteilen sich zu etwa zwei Dritteln auf den SLE (*n* = 859) und zu rund einem Drittel auf die SSc (*n* = 421). Weitere 288 Patienten konnten keiner dieser beiden Kollagenosen zugeordnet werden. Eine Lungenbeteiligung wurde bei Patienten mit SSc deutlich häufiger berichtet als bei Patienten mit SLE (SLE: *n* = 122, 14,2 %; SSc: *n* = 164, 39 %; Abb. 1).

Die Versorgung der Patienten erfolgte durch 54 niedergelassene Praxen sowie 7 Zentren. Der überwiegende Anteil der Patienten wurde in Kliniken bzw. in deren angeschlossenen Ambulanzen betreut (*n* = 1223), während 331 Patienten in der ambulanten vertragsärztlichen Versorgung angebunden waren. Bei 15 Patienten aus 6 Praxen war keine eindeutige Zuordnung möglich.

Der Großteil der Patienten mit SLE und SSc befindet sich derzeit schon in rheumatologischer Behandlung (*n* = 1262, 80,4 %; Tab. 1), eine Lungenbeteiligung wird unter diesen Patienten bei 13,9 % berichtet. Die zweite relevante Fachrichtung stellen die Allgemeinmediziner dar, durch welche 175 (11,2 %) Patienten versorgt werden. 65 Patienten (4,1 %) werden durch Pneumologen betreut, welche erwartungsgemäß alle eine Lungenbeteiligung aufweisen.

**Tab. 1:** Patientenverteilung nach Fachrichtung

Patientenverteilung nach Fachrichtung		
Fachrichtung	Anzahl	Mit Lungenbeteiligung
Allgemeinmediziner	175	21,1 %
Rheumatologen	1262	13,9 %
Pneumologen	65	100,0 %
Nephrologen	27	14,8 %
Internisten	19	10,5 %
Weitere	21	14,3 %
*Summe*	*1569*	*18,2* *%*

Über alle Rückmeldungen hinweg ergab sich ein Durchschnittsalter der Patienten von ca. 52 Jahren. Das Geschlechterverhältnis lag bei 4:1 zugunsten weiblicher Patientinnen (weiblich *n* = 1260, männlich *n* = 302, keine Angabe *n* = 5).

Der überwiegende Teil der rückmeldenden Praxen betreut weniger als 2000 Patienten pro Quartal (*n* = 186), nur 20 Praxen gaben an, dass sie mehr Patienten pro Quartal behandeln, 4 Praxen machten hierzu keine Angabe.

### Hauptsymptome

Bezüglich der Art der pulmonalen Beteiligung handelt es sich in der Mehrzahl der Fälle um eine interstitielle Lungenerkrankung (≈75 %). Etwa die Hälfte der betroffenen Patienten weist eine pulmonale Hypertonie auf, und bei etwas mehr als einem Drittel findet sich eine progrediente Fibrose.

Die am häufigsten berichteten Symptome sind Belastungsdyspnoe (≈90 %) und trockener Husten (≈70 %). Darüber hinaus werden Fatigue (≈63 %), das Raynaud-Syndrom (≈50 %) sowie Myalgien (≈35 %) und Ruhedyspnoe (≈30 %) häufig genannt. Andere Symptome treten demgegenüber deutlich seltener auf.

Differenziert man die bei Diagnosestellung berichteten Symptome nach den drei relevantesten fachärztlichen Disziplinen und fasst alle übrigen unter „Sonstige“ zusammen, zeigt sich, dass die fünf häufigsten Symptome in allen Gruppen, Allgemeinmediziner, Rheumatologen, Pneumologen und sonstige Fachärzten, nahezu identisch sind. Allerdings bestehen teilweise deutliche Unterschiede in der Häufigkeit, mit der die einzelnen Symptome wahrgenommen und berichtet werden. Hierzu gibt Tab. 2 einen Überblick.

**Tab. 2:** Überblick über die berichteten Hauptsymptome beim SLE und der SSc

Symptomatik bei Kollagenosen mit Lungenbeteiligung		
*Erkrankungsart*		
Gesamt	286	100,0 %
SLE	122	42,7 %
SSC	164	57,3 %
*Form der Lungenbeteiligung*		
Interstitielle Lungenerkrankung	213	74,5 %
Pulmonale Hypertonie	133	46,5 %
Progrediente Fibrose	105	36,7 %
*Symptome*		
Belastungsdyspnoe	256	89,5 %
Trockener Husten	201	70,3 %
Fatigue	181	63,3 %
Raynaud-Syndrom	137	47,9 %
Myalgien	101	35,3 %
Ruhedyspnoe	85	29,7 %
Serositis	46	16,1 %
Fieber	24	11,3 %
Neurologische Symptome	14	4,9 %

Die Belastungsdyspnoe wurde durchweg am häufigsten beschrieben, jedoch variiert diese erheblich: Während sonstige Fachärzte diese in 100 % der Fälle angeben, berichten Pneumologen sie nur in etwa 70 % der Fälle. Als zweithäufigstes Symptom wird überwiegend trockener Husten mit knapp über 60 % genannt, bei den sonstigen Fachrichtungen sogar mit 83 %. Eine Ausnahme stellen hierbei die Rheumatologen dar: Sie berichten Fatigue mit etwa 75 % häufiger als trockenen Husten und damit deutlich häufiger als alle anderen Fachrichtungen.

Allgemeinmediziner und die Gruppe der sonstigen Fachrichtungen nennen in absteigender Reihenfolge Myalgien, Ruhedyspnoe und Fatigue als weitere häufige Symptome, wobei diese von Allgemeinmediziner durchweg etwas seltener berichtet werden.

Bei den Pneumologen kehrt sich diese Reihenfolge um. Bemerkenswert ist, dass Ruhedyspnoe sowohl von Pneumologen als auch von Allgemeinmedizinern in etwa 34 % der Fälle angegeben wird; bei den Rheumatologen zählt sie jedoch nicht zu den häufigsten Symptomen und tritt in weniger als einem Drittel der Fälle auf.

Als einzige Fachgruppe berichten Rheumatologen über das Auftreten des Raynaud-Syndroms, das bei etwa zwei Dritteln der Patienten vorkommt und damit an vierter Stelle rangiert. Myalgien werden in dieser Gruppe nur halb so häufig genannt, sodass sie das fünfthäufigste Symptom darstellen.

### Medikamentöse Behandlung, Remission und Therapiezufriedenheit

Die Therapie der Kollagenosen mit Lungenbeteiligung wurde differenziert nach Erkrankungsbild und fachärztlicher Ausrichtung analysiert (Tab. 3).

**Tab. 3:** Rückmeldung nach Fachrichtung

Rückmeldung nach Fachrichtung								
*Symptome*	*Allgemeinmediziner*				*Rheumatologen*			
	Belastungsdyspnoe	82			Belastungsdyspnoe	91		
	Trockener Husten	63			Fatigue	75		
	Myalgien	48			Trockener Husten	69		
	Ruhedyspnoe	34			Raynaud-Syndrom	67		
	Fatigue	28			Myalgien	33		
*Medikation*	*SLE*		*SSc*		*SLE*		*SSc*	
	< 20 mg PÄD/d	44	< 20 mg PÄD/d	50	CYC	98	< 20 mg PÄD/d	74
	MTX	25	MMF	25	< 20 mg PÄD/d	89	MMF	68
	AZA	18	MTX	19	MMF	88	CYC	35
	HCQ/TOC	10	HCQ/RTX/NIN	6	BEL	88	NIN	27
*Remissionsrate*	Immunsuppression	93			Immunsuppression	73		
	GC-Monotherapie	0			Therapiefrei	14		
	Therapiefrei	7			GC-Monotherapie	13		
*Symptome*	*Pneumologen*				*Sonstige*			
	Belastungsdyspnoe	71			Belastungsdyspnoe	100		
	Trockener Husten	61			Trockener Husten	83		
	Fatigue	45			Myalgien	54		
	Ruhedyspnoe	34			Ruhedyspnoe	52		
	Myalgien	34			Fatigue	35		
*Medikation*	*SLE*		*SSc*		*SLE*		*SSc*	
	< 20 mg PÄD/d	40	< 20 mg PÄD/d	40	< 20 mg PÄD/d	88	< 20 mg PÄD/d	58
	NIN	25	NIN	27	MTX	50	MTX	23
	MTX	24	MTX	20	AZA	12	MMF	8
	AZA	10	RTX	14	TOC	10	NIN	7
*Remissionsrate*	Immunsuppression	71			Immunsuppression	47		
	GC-Monotherapie	18			GC-Monotherapie	31		
	Therapiefrei	11			Therapiefrei	22		

Alle Angaben erfolgen in %

*PÄD* Prednisolonäquivalente Dosis, *MTX* Methotrexat, *AZA* Azathioprin, *HCQ* Cyclophosphamid, *TOC* Tociliumab, *MMF* Mycophenolatmofetil, *RTX* Rituximab, *NIN* Nintedanib, *CYC* Antimalariamittel, *GC* Glukokortokoid

Glukokortikoide stellen in allen befragten Fachrichtungen unabhängig von der Grunderkrankung den am häufigsten eingesetzten Therapiebaustein dar. Eine Ausnahme bildet die Rheumatologie: Beim SLE kommt das krankheitsmodifizierende HCQ dort noch häufiger zum Einsatz, nahezu in 100 % der Fälle. Dennoch werden auch in dieser Gruppe GC in rund 90 % der Therapien verwendet.

Im Anschluss folgen MMF und BEL, die jeweils in etwa 87 % der Fälle eingesetzt werden. Es zeigte sich, dass BEL jedoch nahezu ausschließlich innerhalb der Rheumatologie Anwendung findet; in allen anderen Fachrichtungen wird weniger als ein Zehntel der Patienten damit behandelt. Zur Therapie des SLE mit Lungenbeteiligung verordnen Allgemeinmediziner sowie die nichtspezifizierten Fachrichtungen neben GC überwiegend MTX (≈25 % bzw. 50 %), AZA (≈18 % bzw. 12 %) und TOC; jeweils 10 %. Zudem wurde CYC in der Allgemeinmedizin bei etwa 10 % der Patienten eingesetzt. Pneumologen weichen von diesem Muster ab: Nach GC wird dort am zweithäufigsten NIN eingesetzt (≈25 %), gefolgt von MTX (≈24 %) und erst mit deutlichem Abstand AZA.

Die Therapie der SSc mit Lungenbeteiligung zeigt ein deutlich heterogeneres Muster. Neben GC werden am häufigsten MTX, MMF und NIN verordnet. Rheumatologen greifen bei rund zwei Dritteln ihrer Patienten auf MMF zurück, deutlich häufiger als alle anderen Fachrichtungen. Als nächste Fachrichtung erreichen Allgemeinmediziner mit etwa 25 % die zweithöchsten Werte beim Einsatz von MMF; in den übrigen Fachrichtungen liegt der Einsatz bei unter 10 %. HCQ steht bei den Rheumatologen an dritter Stelle (35 %), wird jedoch nur etwa halb so häufig eingesetzt wie MMF. MTX zählt bei Rheumatologen nicht zu den vier häufigsten Medikamenten; in allen anderen Fachrichtungen dagegen wird es bei rund 20 % der Patienten eingesetzt und rangiert bei den nicht näher spezifizierten Fachrichtungen auf Platz zwei. Pneumologen und Allgemeinmediziner setzen außerdem RTX ein, wobei Pneumologen dieses mit 14 % mehr als doppelt so häufig nutzen. Allein der Multikinase-Inhibitor NIN findet als moderneres Medikament bei der SSc mit Lungenbeteiligung eine breite Anwendung über sämtliche Fachrichtungen hinweg: Rund ein Viertel der Patienten wird sowohl in der Rheumatologie als auch in der Pneumologie damit behandelt; bei den sonstigen Fachrichtungen und Rheumatologen ist es zudem das einzige moderne Therapeutikum, das zu den vier häufigsten Therapieoptionen zählt.

In Bezug auf die medikamentöse Versorgung zeigen sich außerdem deutliche Unterschiede zwischen Fachzentren, hierzu zählen Kliniken mit Ambulanzanschluss, und im niedergelassenen Bereich tätigen Ärzten ohne rheumatologischen Schwerpunkt. Insgesamt werden knapp 12 % der Patienten mit Lungenbeteiligung langfristig (> 6 Monate) mit täglichen Glukokortikoiddosen (GCD) von 10 mg oder mehr behandelt. Im niedergelassenen Bereich trifft dies auf fast dreimal so viele Patienten zu (≈28 %). In Fachzentren hingegen machen derart hohe Dosierungen lediglich 1,5 % der langfristigen GC-Therapien aus. Zudem stellt eine kortisonfreie Therapie fast die Hälfte aller Behandlungsformen in Fachzentren dar (44 %), damit sind dort 17 % mehr Patienten kortisonfrei eingestellt als im niedergelassenen Bereich (Tab. 4).

**Tab. 4:** Gegenüberstellung der Angaben zu Glukokortikoiddosen von nicht-rheumatologischen Praxen und rheumatologischen Zentren

Eingesetzte Glukokortikoiddosen zur Langzeitmedikation				
Praxistyp	> 10 mg/d	10–5 mg/d	< 5 mg/d	Kortisonfrei
Gesamt (*N* = 286)	11,9 %	22,6 %	28,2 %	37,2 %
Facharztzentren (*N* = 171)	1,5 %	11,2 %	43,0 %	44,3 %
Niedergelassener Bereich (*N* = 110)	28,2 %	40,0 %	4,8 %	27,0 %

Als Korrelat der in Fachzentren insgesamt niedrigeren GCD bei Kollagenosen mit Lungenbeteiligung zeigt sich dort ein deutlich höherer Anteil an Patienten, die mit Biologika oder anderen DMARDs behandelt werden, verglichen mit dem niedergelassenen Bereich. Zum Einsatz kommen aktuell 4 Wirkstoffe: BEL, NIN, RTX und TOC.

BEL wird in Fachzentren bei einem Großteil der SLE-Patienten mit Lungenbeteiligung eingesetzt (89 %), während es im niedergelassenen Bereich nahezu keine Rolle spielt (0,6 %). Auch RTX wird im ambulanten Bereich deutlich seltener angewendet (SLE ≈5,5 %; SSc ≈8 %). Die Unterschiede fallen jedoch weniger ausgeprägt aus als bei BEL, da in Fachzentren bei sowohl SLE- als auch SSc-Patienten rund ein Fünftel mit RTX behandelt wird (≈20 % bzw. 21 %).

TOC findet bei der Therapie des SLE in rheumatologischen Fachzentren keinerlei Anwendung, während im niedergelassenen Bereich 4,7 % der Patienten damit behandelt werden.

Der Multikinase-Inhibitor NIN wird in beiden Versorgungsebenen nahezu gleich häufig eingesetzt. Im niedergelassenen Bereich wird NIN bei Patienten mit SLE und Lungenbeteiligung sogar häufiger verordnet (≈25 %) als in Fachzentren (≈15 %). Bei der SSc wiederum liegt der Einsatz von NIN in beiden Behandlergruppen bei 28 % der Patienten (Tab. 5).

**Tab. 5:** Einsatz von Biologika oder Nintedanib bei Patienten mit Lungenbeteiligung

Biologikaeinsatz bei Patienten mit Lungenbeteiligung				
Antikörper	Facharztzentren		Niedergelassene Praxen	
	SLE (71)	SSc (100)	SLE (49)	SSc (60)
Belumimab (BEL)	88,7 %	3,5 %	0,6 %	0,0 %
Rituximab (RTX)	19,7 %	21,0 %	5,4 %	8,3 %
Tocilizumab (TOC)	0,0 %	7,0 %	4,7 %	0,8 %
Nintedanib (NIN)	14,8 %	28,0 %	25,5 %	28,0 %

Die in Tab. 3 dargestellten Remissionsraten zeigen, dass Allgemeinmediziner, Rheumatologen und Pneumologen unter immunsuppressiver Therapie Remissionsraten von über 70 % berichten. Die Gruppe der „sonstigen“ fachärztlichen Ausrichtungen weist hingegen unter Immunsuppression nur Remissionsraten von etwas über 45 % auf, dahingegen ist die Remissionsrate unter Glukokortikoid-Monotherapie mit rund 30 % deutlich höher als bei den anderen Facharztgruppen (< 20 %). Gleichzeitig sind bei nicht näher spezifizierten Fachärzten mehr als ein Fünftel der Patienten auch ohne eine Therapie in Remission, ein fast doppelt so hoher Anteil wie in der Rheumatologie und der Pneumologie. In der Gruppe der Allgemeinmediziner ist der Anteil therapiefreier Patienten in Remission nochmals geringer (≈5 %).

Für die subjektive Bewertung der Behandlungseffektivität wurde die Zufriedenheit der behandelnden Ärzte mit der aktuellen Therapie bei Patienten mit Lungenbeteiligung auf einer Skala von 0 % bis 100 % erhoben. Dabei zeigte sich, dass Rheumatologen mit einer durchschnittlichen Zufriedenheit von etwa 50 % ihre Patienten als deutlich besser kontrolliert einschätzen als die übrigen Fachrichtungen, deren Zufriedenheitswerte im Mittel zwischen 10 % und 25 % liegen.

### Bewertung der Versorgungssituation und Verbesserungsvorschläge

Bei der Bewertung der Versorgungssituation von Patienten mit systemischer Sklerose (SSc) bzw. systemischem Lupus erythematodes (SLE) und pulmonaler Beteiligung ergab sich für Rheinland-Pfalz eine durchschnittliche Schulnote von 3,5. Im Saarland wurde die Versorgungssituation mit einer durchschnittlichen Note von 3,3 geringfügig besser eingeschätzt.

In einem Freitextfeld hatten die Befragten die Möglichkeit, Wünsche und Verbesserungsvorschläge zur aktuellen Versorgungssituation von Patienten mit Kollagenosen bzw. rheumatologischen Erkrankungen im Allgemeinen zu äußern. Die Rückmeldungen zeigen ein weitgehend konsistentes Bild: Häufig genannt wurde der Wunsch nach einer schnelleren rheumatologischen Anbindung von Verdachtsfällen sowie nach einer generellen Erhöhung der Zahl rheumatologischer Fachärzte. Gleichzeitig äußerten viele Ärzte den Wunsch, ihre eigenen rheumatologischen Kenntnisse auszubauen, und forderten mehr Fortbildungsangebote im Bereich der Rheumatologie. Insgesamt wird ein deutlicher Bedarf an zusätzlicher rheumatologischer Fachexpertise sichtbar.

## Diskussion

Ziel dieser Erhebung war es, die aktuelle Versorgungssituation von Patienten mit Kollagenosen und Lungenbeteiligung in Rheinland-Pfalz und dem Saarland zu analysieren, um darauf basierend gezielte Ansatzpunkte zur Optimierung der Versorgung dieser häufig hochmorbiden Patienten zu identifizieren. Die Rücklaufquote für ein solches Design zur Abbildung der Versorgungswirklichkeit unterschiedlicher Settings und Facharztrichtungen kann mit knapp 10 % als ausreichend bezeichnet werden. Darüber hinaus ist davon auszugehen, dass ein Großteil der Ärzte, die keine Patienten mit einem SLE oder einer SSc mit Lungenbeteiligung behandeln, den Fragebogen nicht beantworten und retournieren.

Ebenso wurde aus Gründen des Datenschutzes und der Operationalisierbarkeit die Entscheidung getroffen, die behandelnden Ärzte direkt zu befragen. Daher ist nicht auszuschließen, dass Patienten, die interdisziplinär behandelt werden, doppelt erhoben wurden. Durch die Unterschiede der einzelnen Facharztgruppen bezüglich der genutzten Therapien scheint eine Doppelzählung von Patienten jedoch nicht der Regelfall zu sein.

Das von Brinks et al. berichtete Geschlechterverhältnis von 4:1 (weiblich zu männlich) bei Patienten mit SLE deckt sich mit den in dieser Studie erhobenen Daten. Dabei ist jedoch zu berücksichtigen, dass in der vorliegenden Erhebung die Geschlechterverteilung für alle Kollagenosen gemeinsam erfasst wurde und nicht spezifisch für den SLE. Da insbesondere bei der systemischen Sklerose (SSc) ein noch ausgeprägteres Geschlechterungleichgewicht zu Ungunsten der Frauen beschrieben ist (5:1), ist davon auszugehen, dass das Verhältnis von männlichen zu weiblichen Patienten beim SLE in dieser Stichprobe sogar etwas ausgewogener ausfällt [5, 40].

Die häufigsten nicht direkt mit der Lunge assoziierten Symptome waren die Fatigue (≈65 %) und das Raynaud-Syndrom (≈50 %). Myalgien wurden bei etwa einem Drittel der Patienten dokumentiert. Diese Häufigkeiten liegen deutlich unter den Angaben aus der Literatur, in der muskuloskeletale Symptome in einigen Studien mit bis zu 90 % Häufigkeit beschrieben werden [10, 11].

Arthralgien, die ebenfalls zu den nicht direkt lungenabhängigen Symptomen zählen, wurden in diesem Fragebogen nicht erfasst und könnten eine mögliche Erklärung für die beobachtete Differenz darstellen. In einem Freitextfeld, in welchem weitere Symptome angegeben werden konnten, wurden Arthralgien jedoch nicht erwähnt, was darauf hindeutet, dass diese nicht allzu häufig vorkommen oder als weniger einschränkend/bedeutend erachtet werden. Das Raynaud-Phänomen wurde mit fast 67 % von den Rheumatologen häufiger angegeben als im Gesamtdatensatz (≈48 %) und steht im Einklang mit den Ergebnissen anderer Studien [41].

Es fällt auf, dass Fatigue von den Rheumatologen bei nahezu drei Vierteln der von ihnen betreuten Patienten beschrieben wird, was mehr als 30 % über der Häufigkeit liegt, die in anderen Fachrichtungen erfasst wurde. Dies legt die Vermutung nahe, dass bei Nichtrheumatologen dieses Symptom nicht systematisch erfasst wird bzw. die Relevanz in der Rheumatologie durch eigene spezifische Empfehlungen präsenter ist [42].

Dies könnte vor allem darauf zurückzuführen sein, dass Patienten gezielt aufgrund eines spezifischen Hauptsymptoms, wie einer ausgeprägten Dyspnoe, in einer Fachrichtung vorgestellt werden, während fachfremde Symptome nicht weiter untersucht werden. Darüber hinaus sei erwähnt, dass ausdrücklich nach der Begleitsymptomatik bei Patienten mit Lungenbeteiligung gefragt wurde und es daher Unterschiede in der Häufigkeit des Auftretens einzelner Symptome im Vergleich zur Gesamtheit der Kollagenosepatienten geben könnte, von denen ein bedeutender Anteil nicht unter einer Lungenbeteiligung leidet [43].

Welchen Einfluss die unterschiedliche Symptomwahrnehmung auf die Therapieintensivität hat, lasst sich aus den erhobenen Daten nicht ermitteln und bedürfte weiterer Forschung.

Wie bereits beschrieben, stellt HCQ den Grundbaustein der Therapie des systemischen SLE dar und sollte allen Patienten verabreicht werden, sofern keine relevanten Kontraindikationen vorliegen [19]. Lediglich die Rheumatologen gaben an, bei nahezu allen betreuten SLE-Patienten HCQ (98 %) zu rezeptieren.

Glukokortikoide sind noch heute ein fester Bestandteil zur Therapie des SLE, jedoch ist es stets das Ziel, die Dosierung möglichst zügig soweit wie möglich zu reduzieren und optimalerweise darauf zu verzichten [11, 19, 20]. Dennoch wurden 34 % aller durch die befragten Ärzte versorgten Patienten dauerhaft (länger als 6 Monate) höher dosierte (> 5 mg/d) GC rezeptiert. Andererseits erhalten 36 % aller Patienten keine langfristigen GC-Gaben. Dies liegt fern der Daten aus der Kerndokumentation, wonach 56 % der rheumatologisch angebundenen SLE-Patientinnen eine GC-freie Therapie erhielten [44]. Mit etwa 44 % GC-frei eingestellten Patienten nähern sich die Rheumatologen diesem Wert.

Der Anteil rheumatologisch betreuter Patienten, bei denen eine GCD von 5 mg/d oder weniger ausreichend ist, beträgt 42 % und ist damit nur geringfügig höher als bei nicht rheumatologisch betreuten Patienten Die Hauptursachen für diesen Unterschied liegen vermutlich im häufigeren Einsatz von HCQ bei SLE sowie dem generell breiteren Einsatz von Biologika in der Rheumatologie. Besonders Belimumab, welches beim SLE außerhalb der Rheumatologie kaum Anwendung findet, wird von Rheumatologen bei einem Großteil der Patienten eingesetzt. Es sei an dieser Stelle noch angemerkt, dass der Unterschied möglicherweise nur deshalb so gering ausfällt, da in der Rheumatologie kompliziertere und zum Teil therapierefraktäre Fälle behandelt werden, deren adäquate Betreuung in einer anderen Fachrichtung nicht gewährleistet wäre.

Im Hinblick auf die SSc zeigte sich, dass von insgesamt 105 SSc-Patienten mit progredienter Fibrose 43 Patienten das einzige in dieser Indikation zugelassene Medikament, Nintedanib, erhielten. Dies würde bei großzügiger Schätzung eine Abdeckung von etwa 40 % ergeben. Biologika werden aufgrund ihres Off-label-Status entsprechend seltener verordnet [14].

Glukokortikoide kommen insbesondere bei Krankheitsschüben zum Einsatz, wobei höhere Dosen (> 5 mg/d) nur unter strenger Indikationsstellung und sorgfältiger Nutzen-Risiko-Abwägung verordnet werden sollen. Langfristig sollte die tägliche Dosis so gering wie möglich gehalten bzw. ausgeschlichen und potenzielle Komorbiditäten wie die Hypertonie überprüft werden, um das Risiko einer renalen Krise zu reduzieren [22]. Bestenfalls sollte die Dosis 5 mg/d nicht überschreiten [20].

Lediglich die Rheumatologen erzielten einen zufriedenstellend niedrigen Einsatz von Glukokortikoiden. Mögliche Ursachen hierfür könnten schwerwiegendere Krankheitsverläufe in anderen Fachrichtungen aufgrund mangelnder Basistherapien einerseits sowie mehr Erfahrung mit Off-label-Therapien, worunter der Großteil der neuen Medikamente fällt, und deren Beantragung seitens der Rheumatologen andererseits sein. Dies unterstreicht erneut den dringenden Bedarf nach einer höheren Anzahl rheumatologischer Fachärzte, um eine leitliniengerechte Therapie sicherzustellen und eine langfristige Symptomkontrolle zu ermöglichen [2, 14, 19, 22, 25, 45].

Die im Freitextfeld geäußerten Verbesserungsvorschläge konzentrierten sich nahezu ausschließlich auf die Forderung nach schnellerer rheumatologischer Mitbetreuung oder einer allgemeinen Erhöhung der Anzahl rheumatologischer Fachärzte sowie auf Fortbildungsmaßnahmen zur Erweiterung der rheumatologischen Expertise innerhalb anderer Fachrichtungen. In diesem Kontext äußerten 62 % der befragten Praxen eine positive Haltung gegenüber der Idee eines zentralen Netzwerks für rheumatologische Verdachtsfälle. Ein solches Netzwerk wurde bereits mit Rheuma-VOR etabliert und mittlerweile um die Verdachtsdiagnostik einer Kollagenose und im speziellen einer Kollagenose mit Lungenbeteiligung erweitert [46].

Die Empfehlung, eine stärkere Vernetzung in Kombination mit einer verstärkten interdisziplinären Kommunikation zu fördern, findet sich bereits in früheren Studien und zielt darauf ab, eine optimale Therapie sicherzustellen [11, 19, 22, 25]. Hierzu bedarf es, wie im aktuellen Memorandum gefordert, einer Reihe von Maßnahmen und struktureller Veränderungen [45].

Damit einhergehend wird eine Gesamtnote von knapp 3,4, entsprechend einem gerade noch befriedigend, bezüglich der Zufriedenheit mit der aktuellen Versorgungssituation vergeben.

Dies deckt sich mit den bereits 2009 erhobenen Daten von Danoff-Burg und Friedberg zur Zufriedenheit aus Patientensicht. Diese beschreiben, dass nahezu die Hälfte der Befragten das Bedürfnis nach einer engeren rheumatologischen Betreuung haben, sowohl zeitlich als auch in Hinblick auf die Kontakthäufigkeit mit dem Facharzt [47].

Der dringende Bedarf an erweiterten Fortbildungsangeboten, der bereits in der Literatur thematisiert wurde, zeigt sich nicht nur in den Verbesserungsvorschlägen zur Versorgung von Kollagenosepatienten, sondern auch in der Anwendung von zu hohen GCD oder dem unzureichenden Einsatz der empfohlenen Basistherapeutika [48]. Diese Divergenzen zum aktuellen Wissensstand unterstreichen den Bedarf an zusätzlicher Information. Zudem ist eine frühzeitige Diagnose entscheidend, um bleibende Organschäden zu verhindern [49, 50]. Damit diese Diagnose auch gestellt werden kann, ist es allerdings nötig, dass Nicht-Rheumatologen ausreichend mit den Symptomen von Kollagenosen vertraut sind und einen Verdacht frühzeitig äußern können [19, 22].

Um eine leitliniengerechtere Therapie auch durch „Nicht-Spezialisten“ zu ermöglichen und das Interesse sowie die Aufmerksamkeit gegenüber der Rheumatologie und ihrer Krankheitsbilder zu fördern, wäre ein Ansatz, die Präsenz der Rheumatologie in der medizinischen Lehre zu stärken. Bereits 2021 wiesen Aringer et al. auf diese Möglichkeit hin und empfahlen, die Rheumatologie mit mindestens 6 Doppelstunden als Pflichtvorlesung oder als PJ-Wahlfach in das Curriculum aufzunehmen und sicherzustellen, dass dieser Inhalt auch in relevanten Prüfungen ausreichend abgefragt wird [51]. Die Vermittlung grundlegender Kenntnisse zu rheumatologischen Krankheitsbildern im allgemeinmedizinischen Kontext kann eine wesentliche Grundlage zur Verbesserung der Versorgungsqualität rheumatologischer Patientinnen und Patienten schaffen. Insbesondere erscheint es sinnvoll, Hausärztinnen und Hausärzte zur Betreuung unkomplizierter Verläufe, etwa bei rheumatoider Arthritis oder axialer Spondyloarthritis, zu befähigen, um Versorgungskapazitäten effizienter zu nutzen [2, 45, 52]. Ein Grundstein hierzu wurde durch das Disease Management Programm RA gelegt, das es noch adäquat umzusetzen gilt [45].

Zusammenfassend sollten Patienten mit Kollagenosen sowie mit Organ- oder insbesondere Lungenbeteiligung aufgrund der häufig komplexen Krankheitsverläufe primär in spezialisierten rheumatologischen Zentren diagnostiziert und therapiert werden. Für diese Patientengruppe ist darüber hinaus ein interdisziplinärer Behandlungsansatz essenziell. Insbesondere die Betreuung von Patientinnen und Patienten mit Kollagenosen und interstitieller Lungenerkrankung (ILD) sollte fachübergreifend erfolgen, idealerweise im Rahmen eines strukturierten ILD-Boards, um eine leitliniengerechte Therapie sicherzustellen und ein Fortschreiten der Lungenerkrankung bestmöglich zu verhindern.

Inhaltlich als limitierend ist zu erwähnen, dass die Aussagekraft über die meisten Facharztgruppen als gering einzustufen ist, da nur eine sehr begrenzte Anzahl an Praxen eine Rückmeldung gegeben hat. Es ist somit nicht auszuschließen, dass die Unterschiede zwischen Rheumatologen und anderen Fachrichtungen geringer ausfallen.

Auch sei erwähnt, dass durch die Vorlage einer Auswahl an Symptomen seltenere Symptome, die über das Freitextfeld hätten genannt werden müssen, unterrepräsentiert sein können.

Des Weiteren konnte in dieser Umfrage nur die Lungenbeteiligung als mögliche Manifestation der untersuchten Systemerkrankungen genauer betrachtet werden, da der Umfang des Fragebogens inhaltlich und zeitlich begrenzt gehalten werden sollten, um den Rücklauf positiv zu beeinflussen.

Die vorliegenden Ergebnisse der Erhebung zur Versorgungrealität von Patienten mit SLE und SSc mit pulmonaler Beteiligung zeigen deutliche Unterschiede in der Behandlung und Symptomwahrnehmung je nach Fachrichtung sowie Optimierungspotenziale in der interdisziplinären Zusammenarbeit und in der Fortbildung. Die häufige Angabe von Fatigue und des Raynaud-Phänomens insbesondere unter den Rheumatologen verdeutlicht die Notwendigkeit einer spezifischeren Diagnostik und einer stärkeren Einbindung rheumatologischer Expertise, um die jeweilige Systemerkrankung ganzheitlich zu beurteilen.

Es wird ein deutliches Ungleichgewicht in der Glukokortikoidtherapie festgestellt: Während in Fachzentren eine gezieltere Reduktion der Dosen und ein höherer Einsatz von DMARDs zu beobachten sind, werden in niedergelassenen Praxen häufiger – vermutlich aufgrund der vorliegenden Off-label-Situation vieler Medikamente – hohe GCD verordnet. Die verstärkte Nutzung von Biologika oder neu entwickelter gezielter Therapieansätze zur Behandlung der ILD könnte langfristig zur Reduktion der GCD und somit der unerwünschten Nebenwirkungen wie Osteoporose, Gewichtszunahme oder Muskelschwund beitragen.

## Fazit für die Praxis

Zusammenfassend zeigt sich für die Versorgungsrealität, dass eine verstärkte interdisziplinäre Zusammenarbeit, eine stärkere Vernetzung sowie eine verstärkte Fortbildung im Bereich der Rheumatologie notwendig sind, um eine leitliniengerechte Therapie sicherzustellen und die Versorgungsqualität von Patienten mit Kollagenosen und pulmonaler Beteiligung zu verbessern. Eine breitere Implementierung von Biologika bzw. DMARDs allgemein und eine verstärkte Fokussierung auf die Reduktion von Glukokortikoiden könnten dabei helfen, die Behandlungsergebnisse langfristig zu optimieren und Nebenwirkungen zu minimieren.

## Data Availability

Alle dieser Arbeit zugrunde liegenden Daten sind in diesem Artikel enthalten.
